# Association of Tooth Loss with New-Onset Parkinson's Disease: A Nationwide Population-Based Cohort Study

**DOI:** 10.1155/2020/4760512

**Published:** 2020-07-13

**Authors:** Ho Geol Woo, Yoonkyung Chang, Ji Sung Lee, Tae-Jin Song

**Affiliations:** ^1^Department of Neurology, Seoul Hospital, Ewha Womans University College of Medicine, Seoul, Republic of Korea; ^2^Department of Neurology, Kyung Hee University College of Medicine, Seoul, Republic of Korea; ^3^Department of Neurology, Mokdong Hospital, Ewha Womans University College of Medicine, Seoul, Republic of Korea; ^4^Clinical Research Center, Asan Medical Center, Seoul, Republic of Korea

## Abstract

**Introduction:**

Tooth loss is associated with poor oral hygiene. During insufficient oral sanitation, focal infection and inflammation can occur and these reactions may induce systemic inflammation. Systemic inflammatory reaction may be related to the degeneration of dopamine neurons in the substantia nigra. We hypothesized that tooth loss is related to increased risk of new-onset Parkinson's disease.

**Methods:**

Between 2003 and 2006, we included 153,165 participants from the national health insurance system-health screening cohort in Korea. The incidence of new-onset Parkinson's disease was defined as International Classification of Diseases-10 code “G20,” accompanying the prescription records for any anti-Parkinson's disease medication.

**Results:**

Approximately 19.9% of the included participants had periodontal disease. After a median duration of 10.4 years, 1,227 (0.8%) cases of new-onset Parkinson's disease were noted. The number of tooth loss was positively related to an increased risk of new-onset Parkinson's disease. Contrastingly, the frequency of tooth brushings and dental clinic visits for any causes as well as competent dental care were negatively related to the development of new-onset Parkinson's disease. In multivariable analysis, the number of tooth loss (≥15) was positively related to new-onset Parkinson's disease development (hazard ratio: 1.38, 95% confidence interval (1.03–1.85), *p*=0.029, *p* for trend = 0.043) after adjusting variables.

**Conclusion:**

Our study demonstrated that the number of tooth loss was positively correlated with a higher risk of new-onset Parkinson's disease development in a longitudinal study setting. Increased number of tooth loss may be an important risk indicator of new-onset Parkinson's disease.

## 1. Introduction

Parkinson's disease (PD) is an ongoing neurodegenerative disease represented by diverse progressive motor symptoms including postural instability, bradykinesia, rigidity, and tremor [1]. Despite the long-term history of the disease, the etiology and causes of PD have not been well understood. Moreover, only few drugs or treatment methods have been proven effective against loss or damage of the dopamine neuron, which is a fundamental mechanism of PD development [2]. Apparently, the current treatment methods for PD include medical therapy with levodopa, dopamine pathway-targeting drugs, and stereotaxic surgery, which are focused toward achieving only symptomatic relief [3]. However, there is a dearth of disease-modifying agents and preventive methods for PD development. Presumably, genetic factors and environmental factors (head trauma and some pesticides) may lead to the death of dopamine neurons, which in turn result in PD, while consumption of coffee or tea may reduce the risk of PD development [2, 4].

Periodontal diseases are the common oral diseases and tooth loss is frequent condition which encountered in the general population [5]. Periodontal disease is closely associated with an insufficient oral sanitation, such as infrequent tooth brushing and tooth loss [6]. Moreover, previous studies have reported that the frequency of tooth brushings, competent dental care, and number of tooth loss are closely associated with oral hygiene [7–9]. Presence of periodontal diseases, which include gingivitis and periodontitis, is related to inflammatory status affecting the surrounding tissues of the teeth, which may lead to tooth loss and result in systemic inflammation [10]. Periodontitis is the main cause of tooth loss in middle aged and older adults [11]. In previous studies, increased number of tooth loss caused by chronic inflammation due to gingivitis and periodontitis was related to cardiovascular diseases including myocardial infarction, stroke, and long-term mortalities [12–17].

Peripheral and systemic inflammation can aggravate the injury in the brain with neurodegenerative disease [18]. In PD, systemic inflammation has been reported to be chiefly related to microglial activation which is essential for the degeneration of dopamine neurons in the substantia nigra [18, 19]. As insufficient oral sanitation and tooth loss could induce transient bacteremia and systemic inflammation, it is hypothesized that tooth loss would be risk indicator to PD development. We investigated the association of tooth loss with new-onset PD in a longitudinal study setting.

## 2. Materials and Methods

### 2.1. Participants

The National Health Insurance System (NHIS), which obtains demographic information and records regarding the diagnosis and treatment of almost 97% of the Korean population, supplies a government-supported health examination database (National Health Insurance System-National Health Screening Cohort (NHIS-HEALS)) in the present study via random sampling from 2002 to 2015 [20]. Members of the NHIS are proposed routine, biannual health checkups [21]. During health checkups, the weight and height of the individuals were measured and laboratory tests were performed and questionnaires on lifestyle, dental disease status, and oral sanitation practices were administered. Participants aged above 40 years underwent screening programs for dental diseases and insufficient oral sanitation. In case the presence of dental problems was confirmed by dentists, oral health care was proposed to those participants [12].

**Figure 1:**
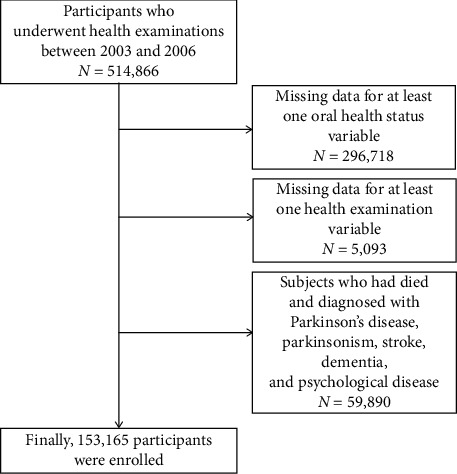
Flowchart of the study participants.

Our study used records of the NHIS-HEALS database collected from 2003 to 2006 for considering a wash-out period of more than 1 year for a new-onset to rule out the possibility of a reverse causal relationship and the presence of untreated PD participants. All the participants underwent routine medical examinations and their medical histories, including age, sex, income level, body mass index, comorbidities, lifestyle habits, such as alcohol intake, smoking status, and regular physical activity, blood pressure, laboratory test results, and oral hygiene indicators, were investigated. Among a total of 514,866 participants, individuals with missing data for variables (*n* = 301,811) were excluded. We excluded individuals (*n* = 59,890) who had died and those diagnosed with PD (International Classification of Diseases, 10^th^ Revision (ICD-10) G20), parkinsonism (ICD-10 G21–26), stroke (ICD-10 I60–I69), or psychological diseases and dementia (ICD-10 F01–F99 and G30–G31) over the past 4 years before the index date. Finally, 153,165 participants were analyzed in the present study (Figure 1).

### 2.2. Study Variables and Definitions

The definition of comorbidities is described in the Supplementary methods. Information regarding oral hygiene behavior (dental clinic visits for any causes, competent dental care, and frequency of tooth brushings) was collected via self-reported data [22]. Presence of periodontal disease was defined as when relevant ICD-10 codes (acute periodontitis (K052), chronic periodontitis (K053), periodontosis (K054), other periodontal disease (K055), and unspecified periodontal disease (K056)) were claimed more than two times by a dentist or when subjects received treatment for periodontal disease with ICD-10 codes (K052-056) by a dentist based on previous studies [12, 23–25]. Dental clinic visits for any causes and competent dental care were dichotomized as never or at least once a year. The frequency of tooth brushings was classified as follows: 0-1 times/day, 2 times/day, and ≥3 times/day. The number of tooth loss, which was ascertained by a dentist, was categorized as 0, 1–7, 8–14, and ≥15 regardless of the reason. We defined the index date as the date of the oral health checkup. If more than two dental health checkups between 2003 and 2006 were undergone by the participants, the most recent data was analyzed.

The incidence of PD was designated as the primary or secondary diagnosis of PD (ICD-10 G20) at least one claim per year by a neurologist, a neurosurgeon, or a rehabilitation medicine specialist added to record of visiting an outpatient clinic or admission accompanying the prescription for anti-PD drug (amantadine, anticholinergics, selegiline, rasagiline, catechol-O-methyltransferase, dopamine agonist, and levodopa/carbidopa) [23]. To exclude the possibility of secondary parkinsonism, participants with both PD (ICD-10 G20) and parkinsonism (ICD-10 G21–26) were excluded for the incidence of PD. We defined the index date as the first prescription of anti-PD medication inferred from relevant ICD-10 codes on claim record. This study was approved by the Institutional Review Board of Ewha Womans University College of Medicine (approval number: Ewha Clinical Trial Center 2018-01-067). Informed consent was waived because retrospective anonymized data were used.

### 2.3. Statistical Analysis

Independent *t*-test and chi-square test were used for comparatively analyzing continuous and categorical variables, respectively. Because statistical power to detect differences is based on sample size, there is the possibility of a false positive using independent *t*-test and chi-square test on data from a large sample size. Therefore, we investigated standardized differences in demographics between the included and excluded participants, age of onset on PD between patients with and without periodontal disease, and laboratory findings among categorized the number of tooth loss as 0, 1–7, 8–14, and ≥15 regardless of the reason and considered standardized differences of >0.1 as noteworthy.

Regression methods of Fine and Gray for competing risk data (death was a competing event for new-onset PD) were used. Hazard ratios (HRs) and 95% confidence intervals (CIs) were investigated. To adjust confounding factors (age, sex, income level, body mass index, comorbidities, and lifestyle habits such as alcohol intake, smoking status, and regular physical activity in model 1; model 1 + blood and urinary laboratory findings and systolic blood pressure in model 2; model 2 + oral hygiene indicators in model 3), three different multivariable regression models were used. To investigate the trends for HR based on the number of tooth loss and frequency of tooth brushings, the *p* value for trend was evaluated. For a better understanding of oral hygiene effects, subgroup analyses were executed within demographics and vascular risk factors. Using a two-sided Wald test in the Cox proportional hazard regression analyses, interaction between oral hygiene indicator and each subgroup was evaluated. All statistical analyses were conducted using SAS software (version 9.2, SAS Institute, Cary, NC, USA). A *p* value of < 0.05 was considered to be statistically significant.

## 3. Results

When demographics were evaluated, elderly individuals, female sex, and lower income level were more frequently observed among the excluded participants (Supplementary Table 1). The mean age of the participants was 52.8 years; 64.3% were men, 23.7% were current smokers, 34.1% had hypertension, 9.7% had diabetes mellitus, and 16.5% had dyslipidemia. More than 15 tooth loss was observed in approximately 1.0% of the participants. Also, 20.0% and 43.9% of the participants had periodontal disease and had visited a dental clinic for any causes, respectively. Moreover, 42.9% of the participants brushed their teeth more than three times per day. Approximately 26.2% of the participants received competent dental care at least once per year (Table 1).

**Figure 2:**
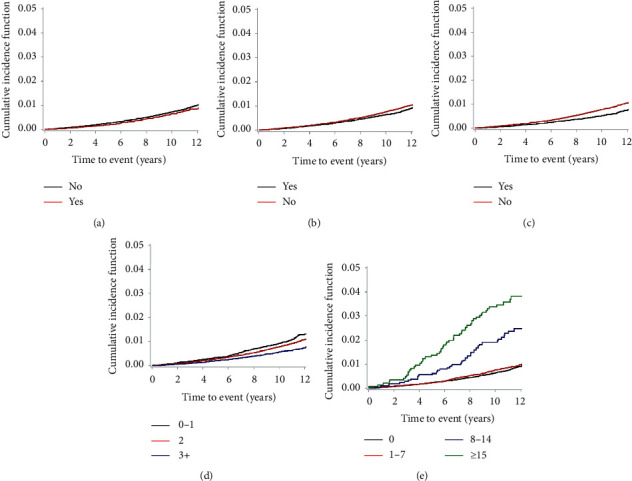
Cumulative incidence curves representing new-onset Parkinson's disease. The cumulative incidence curves for new-onset Parkinson's disease are presented with regard to the following: (a) presence of periodontal disease, (b) dental clinic visits for any causes, (c) competent dental care, (d) frequency of tooth brushings, and (e) number of tooth loss.

With a median duration of 10.4 (interquartile range 9.5–11.7) years, 1,227 cases were diagnosed as new-onset PD. The 10-year event rate was 0.80% for new-onset PD. The cumulative incidence curves for new-onset PD are presented in Figure 2 based on the oral hygiene indicators. The dental clinic visits for any causes (*p*=0.008), competent dental care (*p* < 0.001), and frequency of tooth brushings (*p* < 0.001) were related to a reduced risk of development of new-onset PD. Meanwhile, a large number of tooth loss (≥15) increased the risk of new-onset PD development (*p* < 0.001), whereas the presence of periodontal disease was unrelated to new-onset PD development (*p*=0.140). Association between the presence of periodontal disease and age of onset on PD was shown (*p*=0.082, standardized difference = 0.133) (Supplementary Table 2). Also, total cholesterol and alanine aminotransferase were negatively related to the number of tooth loss and fasting blood glucose level, aspartate aminotransferase, and gamma-glutamyl transferase were positively related to the number of tooth loss (Supplementary Table 3).

In a multivariable analysis, more than fifteen tooth loss were positively related to the development of new-onset PD (HR: 1.33, 95% CI (1.00–1.78), *p*=0.051, *p* for trend = 0.061) after adjusting for age and sex. Furthermore, the number of tooth loss (≥15) remained positively related to the development of new-onset PD in a multivariable analysis (HR: 1.38, 95% CI (1.03–1.85), *p*=0.029, *p* for trend = 0.043) (Table 2). Competent dental care, dental clinic visits for any causes, and frequency of tooth brushings did not show an association with the development of new-onset PD in the multivariable analysis, although oral hygiene indicators were related to new-onset PD development in univariable analysis (Table 2).

In a subgroup analysis, there was no statistically significant interaction between the number of tooth loss and new-onset PD regarding age, sex, alcohol intake, smoking status, regular physical activity, hypertension, diabetes mellitus, and dyslipidemia (Supplementary Table 4).

## 4. Discussion

In the present study, a large number of tooth loss (≥15) represented insufficient oral sanitation, and this correlation can be hypothesized to augment the development of new-onset PD. In a previous study, patients with PD had poorer oral health including taste disturbance, tooth mobility, and chewing/biting problems compared with that of the patients without PD who were matched for age, sex, social background, and lifestyle; apparently, the disease duration was positively related to the number of mobile teeth [26]. Studies on the international literature of case-control study revealed that patients with PD generally had poorer periodontal health such as lesser number of teeth, more dental lesions, and chewing and swallowing difficulties and performed less frequent daily tooth brushings and had limited active mouth opening [27, 28]. Our results are similar to outcomes of previous studies and supply additional information on the association between insufficient oral sanitation and increased development of new-onset PD in a longitudinal study including general population.

In our univariable analysis, indicators of oral hygiene care were negatively related to PD development. Previously, periodontitis and competent dental care were identified as meaningful factors associated with PD in a Taiwanese general population study (odds ratio: 1.431 and 0.204, respectively) [29, 30]. However, the association of indicators of oral hygiene care with the development of PD was not observed in our multivariable analysis. These results suggest that adjusted risk factors in present analysis may be responsible for PD development. In addition, although possibility of relationship between risk factors or associated factors including pesticides, high consumption of dairy products including milk, and caffeine consumption other than adjusted risk factors in the present study and PD development might exist, we cannot adjust these additional factors due to lacked information of NHIS-HEALS in current study [31]. Furthermore, the participants included in our study had a higher income level than those who were excluded. In a previous study, a high income level was associated with a low incidence of poor oral hygiene indicators [32]. The difference in income level between the participants included and excluded in our study may explain the attenuated statistical significance of the association of dental clinic visits for any causes, competent dental care, and frequency of tooth brushings with new-onset PD. In addition, though it was insignificant, paradoxically, our data showed that the presence of periodontal disease was associated with a decreased occurrence of PD. This finding might be explained by the use of anti-inflammatory drugs, such as nonsteroidal anti-inflammatory drugs (NSAIDs), by the subjects with periodontal disease. NSAID is frequently prescribed drugs during the treatment of periodontal disease. Though the results are not consistent, the preventive effect of NSAIDs for PD had been studied and suggested recently [33, 34]. However, it is difficult to say that the effect of NSAIDs use exceeds that of periodontal disease itself on the occurrence of PD. Moreover, the number of subjects who had taken NSAIDs or the amount and frequency of NSAIDs prescription were not evaluated in this study. Another explanation is that the definition of periodontal disease according to ICD-10, as applied in our study, does not incorporate recently published classification criteria and definitions for cases of periodontal disease [35]. Nevertheless, our finding that the number of tooth loss is positively related to the risk of PD development along with the results of our univariable analysis suggest that insufficient oral sanitation is likely to augment the risk of new-onset PD.

Although the current study did not explain the correct mechanism underlying the association of the number of tooth loss with new-onset PD, some hypotheses could account for this association. Insufficient oral sanitation may be related to inflammatory process. It caused tooth loss and ulceration in periodontal pockets that disseminate oral bacteria into the systemic circulation [36]. Moreover, dysbiosis of oral biofilm with highly virulent causes an indirect induction of proinflammatory cytokines [37] which accelerate the inflammation both locally and systemically and affect the brain through humoral and neural pathways. With regard to the humoral pathway, passage of inflammatory cells through the blood–brain barrier that is disrupted because of systemic infection leads to dopaminergic neuronal death via activation of the microglial cells, which produce reactive oxygen species and nitric oxide [38]. In case of the neural pathway, after stimulation of an innate immune reaction by endotoxin lipopolysaccharide (LPS), a cell wall component of oral bacteria, LPS-induced neurotoxicity damages dopaminergic neurons of the substantia nigra of the midbrain [39, 40]. As a surrogate marker of insufficient oral sanitation, the association of a higher number of tooth loss with new-onset PD can be ascribed to these mechanisms.

Our study has some limitations. First, despite its longitudinal nature, the retrospective nature of this study may have caused selection bias, and, thus, direct causal relationships cannot be concluded. Second, because nearly half of the eligible population are excluded from analysis owing to missing data indicators, it could be a significant source of bias. Third, because the information about patient with tooth loss due to either trauma in their youth or dental conditions over the next decades and the information about time of extraction is not acquired from a self-reported questionnaire in National Health Insurance System, we did not know the cause of teeth lost and time of extraction. Fourth, confounding factors including the marital status, educational level, and data about inflammatory state for periodontal disease and blood inflammatory markers were not included because NHIS-HEALS lacked the above-mentioned information. Fifth, our results cannot be generalized to other ethnicities because our dataset included only Korean population. Sixth, because information about some oral hygiene indicators was acquired from a self-reported questionnaire, a recall bias could exist. Seventh, the definition of the presence of periodontal disease based on the ICD-10 code using health claim data does not reflect recently published case-definitions and classification criteria for periodontal disease [35]. Therefore, further study using recently updated periodontal disease classification is needed. Eighth, although we considered a wash-out period, the number of tooth loss could still be a result of motor and nonmotor symptoms that predate the diagnosis of PD.

## 5. Conclusion

Our study demonstrated that the number of tooth loss was related to an increased risk of new-onset PD development and that the number of tooth loss (≥15) may be an augmenting risk indicator of new-onset PD.

## Figures and Tables

**Table 1:** Baseline characteristics of the study population.

Characteristics	Total
Number of participants	153,165
Age (years)	52.8 ± 8.3
Male sex	98,434 (64.3)
Income level	
Fifth quintile (highest)	62,799 (41.0)
Fourth quintile	30,365 (19.8)
Third quintile	21,386 (14.0)
Second quintile	18,872 (12.3)
First quintile (lowest)	19,479 (12.7)
Covered by medical aid	264 (0.2)
Body mass index (kg/m^2^)	23.9 ± 2.8
Alcohol intake	73,523 (48.0)
Smoking status	
Nonsmoker	100,075 (65.3)
Ex-smoker	16,721 (10.9)
Current smoker	36,369 (23.7)
Regular physical activity	15,062 (9.8)
Comorbidities	
Hypertension	52,257 (34.1)
Diabetes mellitus	14,799 (9.7)
Dyslipidemia	25,234 (16.5)
Renal disease	328 (0.2)
History of malignancy	15,337 (10.0)
Blood pressure	
Systolic blood pressure (mmHg)	125.8 ± 16.6
Diastolic blood pressure (mmHg)	78.8 ± 10.8
Laboratory findings	
Total cholesterol (mg/dL)	197.8 ± 36.1
Fasting blood glucose level (mg/dL)	97.9 ± 27.8
Aspartate aminotransferase (U/L)	26.6 ± 16.1
Alanine aminotransferase (U/L)	25.9 ± 20.2
Gamma-glutamyl transferase (U/L)	40.0 ± 56.1
Proteinuria (≥1 + in dip stick test)	4,948 (3.2)
Oral health status	
Presence of periodontal disease	30,580 (20.0)
Number of tooth loss	
0	115,483 (75.4)
1–7	34,077 (22.2)
8–14	2,147 (1.4)
≥15	1,458 (1.0)
Oral hygiene care	
Dental clinic visits for any causes	67,211 (43.9)
Frequency of tooth brushings (times/day)	
0-1	21,382 (14.0)
2	66,089 (43.1)
≥3	65,694 (42.9)
Competent dental care	40,177 (26.2)

Data are expressed as the mean ± standard deviation or *n* (%).

**Table 2:** Risk of new-onset Parkinson's disease according to oral health disease and oral hygiene care.

	Event rate (%), (95% CI)	Unadjusted model		Multivariable adjusted (1)		Multivariable adjusted (2)		Multivariable adjusted (3)	
		HR (95% CI)	*p* value	HR (95% CI)	*p* value	HR (95% CI)	*p* value	HR (95% CI)	*p* value
Presence of periodontal disease									
No	0.79 (0.74, 0.84)	1 (ref)	0.125	1 (ref)	0.104	1 (ref)	0.103	1 (ref)	0.232
Yes	0.68 (0.59, 0.77)	0.89 (0.77–1.03)		0.88 (0.76–1.03)		0.88 (0.76–1.03)		0.91 (0.78–1.06)	

Frequency of tooth brushings (times/day)									
0-1	1.05 (0.93, 1.20)	1 (ref)	0.014	1 (ref)	0.793	1 (ref)	0.775	1 (ref)	0.652
2	0.86 (0.79, 0.93)	0.83 (0.71–0.96)		1.02 (0.88–1.19)		1.02 (0.88–1.19)		1.04 (0.89–1.21)	
≥3	0.59 (0.53, 0.65)	0.58 (0.50–0.69)	<0.001	0.93 (0.78–1.10)	0.370	0.93 (0.78–1.10)	0.384	0.95 (0.80–1.12)	0.531
*p* for trend		<0.001		0.267		0.277		0.399	

Dental clinic visits for any causes									
No	0.83 (0.77, 0.89)	1 (ref)	0.008	1 (ref)	0.169	1 (ref)	0.180	1 (ref)	0.640
Yes	0.70 (0.64, 0.76)	0.86 (0.76–0.96)		0.92 (0.82–1.04)		0.92 (0.82–1.04)		0.97 (0.85–1.11)	

Competent dental care									
No	0.84 (0.79, 0.89)	1 (ref)	<0.001	1 (ref)	0.136	1 (ref)	0.139	1 (ref)	0.436
Yes	0.57 (0.50, 0.65)	0.69 (0.60–0.80)		0.90 (0.78–1.04)		0.90 (0.78–1.04)		0.94 (0.79–1.11)	

Number of tooth loss									
0	0.70 (0.65, 0.75)	1 (ref)	0.083	1 (ref)	0.789	1 (ref)	0.754	1 (ref)	0.765
1–7	0.80 (0.71, 0.90)	1.13 (0.98–1.29)		0.98 (0.86–1.12)		0.98 (0.85–1.12)		0.98 (0.85–1.12)	
8–14	2.20 (1.66, 2.92)	2.94 (2.20–3.93)	<0.001	1.34 (0.99–1.80)	0.055	1.33 (0.99–1.79)	0.059	1.32 (0.98–1.78)	0.068
≥15	3.77 (2.88, 4.94)	4.76 (3.60–6.29)	<0.001	1.40 (1.05–1.87)	0.022	1.39 (1.04–1.86)	0.025	1.38 (1.03–1.85)	0.029
*p* for trend		<0.001		0.032		0.038		0.043	

Event rates were reported as 10-year event rates (%). Regression methods of Fine and Gray for competing risk data (death is a competing event for Parkinson's disease) were used. Multivariable model (1) was adjusted for age, sex, income level, regular physical activity, alcohol intake, smoking status, body mass index (kg/m^2^), hypertension, diabetes mellitus, dyslipidemia, renal disease, and history of malignancy. Multivariable model (2) was adjusted for the variables listed above as well as for systolic blood pressure, fasting blood glucose level, aspartate aminotransferase, alanine aminotransferase, gamma glutamyl transferase, and proteinuria. Multivariable model (3) was adjusted for the variables listed above as well as for the presence of periodontal disease, frequency of tooth brushings, dental clinic visits for any causes, competent dental care, and the number of tooth loss except regarding the independent variable. CI, confidence interval and HR, hazard ratio.

## Data Availability

The data that support the findings of this study are available from NHIS-HEALS, but restrictions apply to the availability of these data, which were used under license for the current study and, hence, are not publicly available. Data are, however, available from the corresponding author upon reasonable request and with permission from the National Health Insurance System.

## Supplementary Materials

Supplementary Materials Supplementary Table 1: baseline demographics between the included and excluded participants in the present study. Supplementary Table 2: association between the presence of periodontal disease and age of onset on Parkinson's disease. Supplementary Table 3: association between laboratory findings and number of tooth loss. Supplementary Table 4: subgroup analysis of the relationship between the number of tooth loss and new-onset Parkinson's disease.
