# Supplementary material for: Association of Tooth Loss with New-Onset Parkinson's Disease: A Nationwide Population-Based Cohort Study
Source: Parkinsons Dis. 2020 Jul 13;2020:4760512. doi: 10.1155/2020/4760512 (PMC7374233; doi:10.1155/2020/4760512)
Supplement: Supplementary Materials — Supplementary Table 1: baseline demographics between the included and excluded participants in the present study. Supplementary Table 2: association between the presence of periodontal disease and age of onset on Parkinson's disease. Supplementary Table 3: association between laboratory findings and number of tooth loss. Supplementary Table 4: subgroup analysis of the relationship between the number of tooth loss and new-onset Parkinson's disease. [file 4760512.f1.pdf]

Supplementary Material Methods

The definition of comorbidity

Information on smoking status and alcohol intake were obtained by questionnaire. Body mass index was calculated as the body weight (in kilograms) divided by the square of the height (in meters). Information regarding alcohol intake and smoking status was acquired using a questionnaire. Smoking status was categorized as non-smoker, ex-smoker, and current smoker. Current smoker was considered as a person who has smoked >5 packs (100 cigarettes) in a lifetime, as defined by the World Health Organization, and has smoked daily or occasionally for the last 28 days. Ex-smoker was considered as a person who has smoked >100 cigarettes in a lifetime but has not smoked over the last 28 days [1]. Regular physical activity was defined as strenuous exercise performed for >20 min and more than once a week [2]. Presence of hypertension, diabetes mellitus, and dyslipidemia was defined using ICD-10 codes and the existence of prescriptions claimed more than once over a year (hypertension: I10–11, diabetes mellitus: E10–14, dyslipidemia: E78). Presence of renal disease and malignancy was defined by ICD–10 codes identified at least once a year (renal disease: N18.1~.5, N18.9, malignancy: C00–D48) [3].

References

1. Lee KH, Lee CM, Kwon HT, Oh S-W. Relationship between Obesity and Smoking in Korean Men: Data Analyses from the Third and Fourth Korea National Health and Nutrition Examination Surveys (KNHANES). JKSRNT. 2010;1(2):115-23. Epub 07/15. doi: 10.25055/JKSRNT.2010.1.2.115.

2. Kim MK, Han K, Joung HN, Baek KH, Song KH, Kwon HS. Cholesterol levels and development of cardiovascular disease in Koreans with type 2 diabetes mellitus and without pre-existing cardiovascular disease. Cardiovasc Diabetol. 2019;18(1):139. doi:

10.1186/s12933-019-0943-9. PubMed PMID: 31640795; PubMed Central PMCID: PMCPMC6805335.

3. Park SY, Kim SH, Kang SH, Yoon CH, Lee HJ, Yun PY, et al. Improved oral hygiene care attenuates the cardiovascular risk of oral health disease: a population-based study from Korea. Eur Heart J. 2019;40(14):1138-45. doi: 10.1093/eurheartj/ehy836. PubMed PMID: 30561631.

Supplementary Table 1. Baseline demographics between the included and excluded participants in the present study

Characteristics	Included participants	Excluded participants	p value	Standardized Difference
Number of participants	153,165	361,701		
Age (years)	**52.8 ± 8.3**	**57.6 ± 9.9**	**<0.001**	**0.530**
Male sex	**98,434 (64.3)**	**180,691 (50.0)**	**<0.001**	**-0.292**
Income level			**<0.001**	**0.258**
Fifth quintile (highest)	**62,799 (41.0)**	**113,581 (31.4)**		
Fourth quintile	**30,365 (19.8)**	**75,684 (20.9)**		
Third quintile	**21,386 (14.0)**	**60,814 (16.8)**		
Second quintile	**18,872 (12.3)**	**53,390 (14.8)**		
First quintile (lowest)	**19,479 (12.7)**	**51,514 (14.2)**		
Covered by medical aid	**264 (0.2)**	**6,718 (1.9)**		

Data are expressed as the mean ± SD, or n (%)

Supplementary Table 2. Association between the presence of periodontal disease and age of onset on Parkinson's disease

	Presence of periodontal disease		p value	Standardized difference
	No	Yes		
Number of patients	1,020	207		
Age of onset on Parkinson's disease	**69.1±9.3**	**67.8±9.3**	**0.082**	**0.133**

Data are expressed as the mean ± SD
p value by Student's t-test

Supplementary Table 3. Association between laboratory findings and number of tooth loss

Laboratory findings	Number of tooth loss				p value	Standardized difference
	0	1-7	8-14	≥15		
TC (mg/dL)	**197.9±36.0**	**197.5±36.5**	**196.8±39.2**	**193.2±37.8**	**<.001**	**0.1296**
FBS (mg/dL)	**97.2±26.5**	**99.8±30.3**	**102.4±39.7**	**100.5±37.6**	**<.001**	**0.1549**
AST (U/L)	**26.3±15.5**	**27.4±17.2**	**28.4±18.9**	**28.9±23.9**	**<.001**	**0.1306**
ALT (U/L)	**25.7±20.1**	**26.7±20.5**	**25.2±18.2**	**23.9±19.8**	**<.001**	**0.1375**
GTP (U/L)	**38.7±53.1**	**44.2±62.7**	**45.8±79.0**	**40.4±73.5**	**<.001**	**0.1058**
Proteinuria (≥1+ in dip stick test)	**3,655 (3.2)**	**1,170 (3.4)**	**63 (2.9)**	**60 (4.1)**	**0.016**	**0.0641**

Data are expressed as the mean ± SD, or n (%)
p value by ANOVA and Chi-square test

TC, Total cholesterol; FBS, Fasting blood glucose level; AST, Aspartate aminotransferase; ALT, Alanine aminotransferase; GTP, Gamma glutamyl transferase

Supplementary Table 4. Subgroup analysis of the relationship between the number of tooth loss and new-onset Parkinson's disease

	Model1		Model2		Model3	
	HR (95% CI)	p value for interaction effect	HR (95% CI)	p value for interaction effect	HR (95% CI)	p value for interaction effect
Age		0.346		0.355		0.343
≤50						
Number of tooth loss						
0	1 (ref)		1 (ref)		1 (ref)	
1-7	0.79 (0.63-1.19)		0.77 (0.51-1.16)		0.78 (0.63-1.17)	
8-14	**2.80 (1.31-8.99)**		**2.52 (1.33-8.06)**		**2.37 (1.30-8.92)**	
≥15	**2.93 (1.14-9.53)**		**2.67 (1.13-9.27)**		**2.73 (1.53-9.44)**	
≥51						
Number of tooth loss						
0	1 (ref)		1 (ref)		1 (ref)	
1-7	1.05 (0.91-1.22)		1.04 (0.89-1.16)		1.04 (0.89-1.15)	
8-14	**1.95 (1.45-2.56)**		**1.93 (1.42-2.49)**		**1.93 (1.43-2.51)**	
≥15	**3.05 (2.30-4.04)**		**2.96 (2.24-3.92)**		**2.82 (2.13-3.74)**	
Sex		0.912		0.952		0.963
Men						
Number of tooth loss						
0	1 (ref)		1 (ref)		1 (ref)	
1-7	0.98 (0.82-1.14)		0.97 (0.82-1.15)		0.98 (0.81-1.15)	
8-14	1.42 (0.92-2.12)		1.41 (0.94-2.11)		1.42 (0.94-2.12)	
≥15	**2.33 (1.11-2.55)**		**2.37(1.08-2.39)**		**2.32 (1.09-2.46)**	
Women						
Number of tooth loss						
0	1 (ref)		1 (ref)		1 (ref)	
1-7	1.02 (0.82-1.27)		1.01 (0.85-1.28)		1.01 (0.83-1.26)	
8-14	1.40 (0.92-2.13)		1.36 (0.89-2.06)		1.34 (0.88-2.08)	
≥15	1.45 (0.94-2.17)		1.44 (0.98-2.10)		1.45 (0.98-2.13)	
Alcohol intake		0.330		0.343		0.354
No						
Number of tooth loss						
0	1 (ref)		1 (ref)		1 (ref)	
1-7	1.05 (0.89-1.22)		1.04 (0.88-1.22)		1.05 (0.89-1.22)	
8-14	1.22 (0.85-1.78)		1.22 (0.83-1.71)		1.22 (0.84-1.71)	
≥15	**1.48 (1.08-2.18)**		**1.48 (1.07-2.08)**		**1.48 (1.06-2.06)**	

Yes						
Number of tooth loss						
0	1 (ref)		1 (ref)		1 (ref)	
1-7	0.92 (0.73-1.14)		0.89 (0.71-1.12)		0.90 (0.72-1.13)	
8-14	**1.82 (1.15-2.89)**		**1.83 (1.15-2.90)**		**1.83 (1.14-2.89)**	
≥15	2.69 (0.97-2.85)		2.64 (0.96-2.80)		2.64 (0.96-2.74)	
Current smoker		0.746		0.755		0.737
No						
Number of tooth loss						
0	1 (ref)		1 (ref)		1 (ref)	
1-7	0.94 (0.83-1.14)		0.97 (0.82-1.13)		0.96 (0.82-1.11)	
8-14	**1.42 (1.03-1.96)**		**1.44 (1.04-1.91)**		**1.37 (1.01-1.91)**	
≥15	**1.58 (1.13-2.14)**		**1.57 (1.13-2.13)**		**1.56 (1.13-2.12)**	
Yes						
Number of tooth loss						
0	1 (ref)		1 (ref)		1 (ref)	
1-7	1.12 (0.84-1.56)		1.12 (0.82-1.53)		1.14 (0.83-1.52)	
8-14	1.28 (0.61-2.70)		1.27 (0.61-2.69)		1.26 (0.60-2.68)	
≥15	2.37 (0.63-2.89)		2.36 (0.60-2.91)		2.32 (0.60-2.93)	
Regular physical activity		0.745		0.746		0.741
No						
Number of tooth loss						
0	1 (ref)		1 (ref)		1 (ref)	
1-7	0.96 (0.84-1.13)		0.97 (0.84-1.13)		0.96 (0.84-1.13)	
8-14	1.32 (0.94-1.75)		1.29 (0.94-1.76)		1.28 (0.93-1.76)	
≥15	**1.52 (1.12-2.08)**		**1.50 (1.11-2.07)**		**1.52 (1.10-2.06)**	
Yes						
Number of tooth loss						
0	1 (ref)		1 (ref)		1 (ref)	
1-7	1.03 (0.72-1.51)		1.03 (0.69-1.47)		1.02 (0.69-1.47)	
8-14	**2.05 (1.01-4.10)**		**2.04 (1.01-4.11)**		2.02 (1.00-4.06)	
≥15	2.76 (0.78-3.75)		2.68 (0.76-3.72)		2.71 (0.74-3.73)	
Hypertension		0.675		0.687		0.696
No						
Number of tooth loss						
0	1 (ref)		1 (ref)		1 (ref)	
1-7	0.99 (0.84-1.16)		0.97 (0.84-1.14)		0.97 (0.84-1.12)	

8-14	**1.46 (1.11-2.24)**		**1.52 (1.11-2.07)**		**1.50 (1.10-2.05)**	
≥15	**1.83 (1.13-2.78)**		**1.94 (1.10-2.26)**		**1.98 (1.09-2.24)**	
Yes						
Number of tooth loss						
0	1 (ref)		1 (ref)		1 (ref)	
1-7	1.02 (0.73-1.38)		1.01 (0.73-1.38)		1.01 (0.73-1.43)	
8-14	1.32 (0.34-1.49)		1.31 (0.35-1.52)		1.31 (0.34-1.54)	
≥15	2.34 (0.84-3.13)		2.32 (0.84-3.11)		2.31 (0.83-3.11)	
Diabetes mellitus		0.565		0.567		0.567
No						
Number of tooth loss						
0	1 (ref)		1 (ref)		1 (ref)	
1-7	0.99 (0.84-1.16)		0.97 (0.84-1.14)		0.97 (0.84-1.12)	
8-14	**1.56 (1.13-2.19)**		**1.52 (1.11-2.07)**		**1.50 (1.10-2.05)**	
≥15	**1.93 (1.11-2.28)**		**1.94 (1.10-2.26)**		**1.98 (1.09-2.24)**	
Yes						
Number of tooth loss						
0	1 (ref)		1 (ref)		1 (ref)	
1-7	1.02 (0.73-1.38)		1.01 (0.73-1.38)		1.01 (0.73-1.40)	
8-14	0.82 (0.34-1.89)		0.81 (0.35-1.90)		0.81 (0.34-1.89)	
≥15	2.64 (0.84-3.17)		2.62 (0.84-3.16)		2.61 (0.83-3.18)	
Dyslipidemia		0.987		0.981		0.981
No						
Number of tooth loss						
0	1 (ref)		1 (ref)		1 (ref)	
1-7	0.98 (0.86-1.18)		0.98 (0.84-1.17)		0.98 (0.85-1.18)	
8-14	1.36 (1.00-1.92)		1.36 (0.99-1.88)		1.35 (0.98-1.89)	
≥15	**2.02 (1.17-2.13)**		**2.03 (1.15-2.12)**		**1.99 (1.14-2.12)**	
Yes						
Number of tooth loss						
0	1 (ref)		1 (ref)		1 (ref)	
1-7	0.97 (0.71-1.32)		0.96 (0.70-1.31)		0.96 (0.71-1.31)	
8-14	1.45 (0.73-2.89)		1.45 (0.73-2.92)		1.46 (0.73-2.92)	
≥15	2.32 (0.60-2.89)		2.32 (0.62-2.93)		2.32 (0.61-2.92)	

Multivariable model (1) was adjusted for age, sex, income level, regular physical activity, alcohol intake, smoking status, body mass index (kg/m2), hypertension, diabetes mellitus, dyslipidemia, renal disease, and history of malignancy except independent variable regarding subgroup

Multivariable model (2) was adjusted for the variables listed above as well as systolic blood pressure, fasting blood glucose level, aspartate aminotransferase, alanine aminotransferase, gamma glutamyl transferase, and proteinuria except independent variable regarding subgroup

Multivariable model (3) was adjusted for the variables listed above as well as presence of periodontal disease, frequency of tooth brushings, dental clinic visits for any causes, competent dental care, and the number of tooth loss except independent variable regarding subgroup
